# Complications of bone transport technique using the Ilizarov method in the lower extremity: a retrospective analysis of 282 consecutive cases over 10 years

**DOI:** 10.1186/s12891-020-03335-w

**Published:** 2020-06-06

**Authors:** Yanshi Liu, Maimaiaili Yushan, Zhenhui Liu, Jialin Liu, Chuang Ma, Aihemaitijiang Yusufu

**Affiliations:** 1 Department of Microrepair and Reconstruction, The First Affiliated Hospital of Xinjiang Medical University, Urumqi, Xinjiang China; 2 Department of Prosthodontics, The First Affiliated Hospital of Xinjiang Medical University, Urumqi, Xinjiang China

**Keywords:** Bone defect, Bone transport, Complication, External fixation

## Abstract

**Background:**

The treatment of large bone defects in lower limbs is a serious challenge for orthopedic surgeons and patients. The bone transport technique using the Ilizarov method has become the main treatment option for the reconstruction of bone defect. However, inevitable difficulties and complications related to bone transport technique have been reported by many studies. The purpose of this study was to evaluate the effectiveness and complications of bone transport technique using Ilizarov method in the treatment of bone defect of lower extremity.

**Methods:**

The study was conducted on 282 patients who underwent bone transport procedures using Ilizarov method at our institution from January 2007 to June 2017. Patient’s demographic data, complications and clinical outcomes at minimum of 2 years follow-up were collected and retrospectively analyzed. All difficulties that related to bone transport were documented according to Paley’s classification. The clinical outcomes were evaluated using Association for the Study and Application of the Method of Ilizarov criteria (ASAMI) at last clinical visit.

**Results:**

There were 243 male and 39 females with a mean age of 40 years (range 18–65 years). The mean defect was 6.56 ± 2.15 cm, whereas single level transport in 221 cases and double level transport in 61 cases. There were 189 problems, 166 obstacles and 406 complications (257 minor and 149 major complications), and the average complication rate per patients consists of 0.91 minor and 0.53 major complications. The top five complications were pin-site infection (65.96%), axial deviation (40.78%), joint stiffness (23.76%), soft tissue incarceration (22.34%) and delayed union of the docking site (13.48%).The ASAMI bony result was excellent in 233 patients, good in 32, fair in 5 and poor in 12. The ASAMI functional result was excellent in 136 patients, good in 88, fair in 47, poor in 11.

**Conclusion:**

Bone transport is a reliable method for reconstruction of bone defects in the femur and tibia. Awareness of predictable complications is beneficial to prevent or early detection of the expected complication which can improve the risk-benefit balance.

## Background

The treatment of large bone defects caused by trauma, developmental deformities, resection of tumor or osteomyelitis in lower limbs is a serious challenge for orthopedic surgeons and patients [1, 2]. Many surgical procedures have been proposed for the treatment of bone defect [3–6], and bone transport technique using the Ilizarov method is widely practiced in reconstructive surgery [7–10]. The Ilizarov method is based on the biology of the bone and the ability of the surrounding soft tissues to regenerate under tension stress, so it become the main treatment option for the reconstruction of bone defect due to it is characterized by rapid, simple, effective and minimally invasive, which can preserve the biomechanical microenvironment needed for fracture healing [9, 11–14].

Although the treatment of bone defect using bone transport technique has been used widely include reconstruction of the bone defect along with soft-tissue coverage, correction of the joint contractures or malalignment. However, inevitable difficulties and complications related to bone transport technique using the Ilizarov method have been reported by many studies [9, 13–19]. It is remaining a major concern to these complications which could affect on the clinical outcomes.

Therefore, the purpose of our study was to retrospectively analyze and evaluate the bone transport-related complications of patients in our institution over the past 10 years in order to predict the risk and related factors of complications.

## Methods

### Patients

This retrospectively study included 282 patients who underwent bone transport procedures in lower extremity using the Ilizarov method at our institution from Jan 2007 to June 2017, including 243 male and 39 females with a mean age of 40 years (range 18–65 years). Patients older than 18 years with bone defect more than 3 cm in the lower extremity were included. Patients with bone defect caused by pathological fractures, associated vascular and nerve injury, age > 65 years, poor compliance, and any other illness that can affect bone healing (such as diabetes, hypertension, osteoporosis, kidney disease, etc.) were excluded. This study was approved by the Ethical Committee of our institution.

### Surgical technique

A complete removal of hardware, radical debridement of all necrotic and infected bone and soft tissue, and/or implantation of an antibiotic-impregnated cement spacer to improve stability were performed prior to bone transport. Cortical bleeding, described as the so-called “paprika sign”, was accepted as an indication of vital osseous tissue [20]. Specimens were taken and sent for bacterial culture and drug susceptibility tests to guide the surgeon for the appropriate postoperative antibiotics. Local tissue flap or directly suture without tension was performed to reconstruct the small soft tissue defects, whereas flap transfer or free skin grafting was used to cover the larger wound.

Bone transport was initiated when clinical manifestations and laboratory indicators showed the infective process had resolved. Preoperative anteroposterior and lateral X-rays were used to evaluate the defect size and plan the construction of the external fixator. The type of external fixator was comprehensively determined by the location of bone and soft tissue defect along with surgeon’s experience and patient’s acceptance. The osteotomy was performed using minimally invasive fashion using Gigli saw technique and special care was given to preserve as much periosteum as possible. Bone defect larger than 8 cm or exceeded 40% of the injured bone underwent a double level bone transport [21–23] procedure. All the procedures were conducted by the same surgical team.

### Data collection

The demographic data include age, sex, weight and height (BMI = weight (kg) /height (m^2^)), injured bone, location of bone defect (proximal, middle and distal), defect size (DS), type of external fixation (circular (TrueLok Ring Fixation System, Orthofix, Verona, Italy) or monolateral (Limb Reconstruction System, LRS, Orthofix, Verona, Italy)), type of bone transport (single level and double level), direction of bone transport (from proximal to distal or distal to proximal in single level and converging or “twin” transport in double level) were collected.

Postoperative data were recorded include distraction regenerate length (DRL), docking time (DT), regenerate consolidation time (CT), external fixation time (EFT/ET), external fixation index (EFI) and type of difficulties occurred during and after bone transport procedure. The EFT referred to the time spend on before removal of the external fixator. The EFI was defined as the ratio of the days of EFT to the DRL (centimeters). Radiographic evaluation was conducted every 2 weeks during the bone transport period and monthly in the consolidation phase. All patients were closely followed up at minimum of 2 years after the removal of external fixator.

According to Paley [24], difficulties that occur during limb lengthening were subclassified into problems, obstacles and complications. A problem is defined as a potential expected difficulty that arises during the distraction or fixation period that is fully resolved by the end of the treatment period by nonoperative means. An obstacle is defined as a potential expected difficulty that arises during the distraction or fixation period that is fully resolved by the end of the treatment period by operative means. Complications include any local or systemic intraoperative or perioperative complication, a difficulty during distraction or fixation that remains unresolved at the end of the treatment period, and any early or late posttreatment difficulty. True complications were subclassified as minor and major complications. Minor complications did not affect the final result or required nonoperative or a minor operative intervention, while major complications required a more complex and unplanned operative intervention or resulted in permanent sequelae. The bone and functional results were assessed by ASAMI [21] at the last clinical visit.

### Postoperative management

All patients were encouraged to do isometric muscle and joint range of motion (ROM) exercise within the tolerance of pain on the second day after surgery. Antibiotics that are suitable according to the results of cultures and antibiotic susceptibility tests are applied intravenously for at least 3 weeks or until the erythrocyte sedimentation rate (ESR) and C-reactive protein (CRP) levels return to normal limits.

According to the reference data [25, 26], after latency period of 7 to 10 days, bone transport started at a rate of 1 mm (single level) or 2 mm (double level) daily, 4 times a day. The rate of bone transport was adjusted according to patients’ tolerance and the quality of the regenerate. The procedure of bone transport continued for 4 or 5 days to compress the docking site after the docking. The external fixator was dynamized before removal. And removal of the external fixator was conducted when the standard orthogonal radiographs showed sufficient consolidation of the distraction zone (dense bone formation) and solid docking site union (corticalization in 3 of 4 cortices). Additionally, all patients were put on the functional brace for 4–6 weeks for the protection of refracture.

### Statistical analysis

Statistical analysis was performed with the SPSS 22.0 (IBM Corp, USA) and R Studio (Version 1.2.5001) with rms, ROCR, gplots and forest plot packages. Continuous variables were analyzed by Independent-samples T tests and expressed as the mean and standard deviation. And the count variables were analyzed by the Chi-square or Fisher’s test, expressing as number. Statistically significant difference was set at *P* < 0.05.

Variables with *P* < 0.05 were identified in the univariate logistic regression analysis (ULRA). And the variables with *P* < 0.1 in the ULRA were included in the multivariate logistic regression analysis (MLRA), then variables with *P* < 0.05 were screened out by the stepwise method and these variables were used to construct the nomograms. Finally, the ability of nomograms to distinguish the models was evaluated by the area under the curve (AUC) of the receiver operating characteristic (ROC) curve.

## Results

The etiology of bone defect includes posttraumatic in 97 cases (17 femur and 80 tibias), osteomyelitis in 146 (31 femurs and 115 tibias), infected nonunion in 26 (11 femurs and 15 tibias) and atrophic nonunion in 13 (3 femurs and 10 tibias). A total of 220 tibial and 62 femoral bone transport procedures in our study were collected. The Ilizarov circular fixator was applied in 128 cases (all in tibia), and the monolateral fixator was applied in 154 cases (62 femurs,92 tibias). The average defect size was 6.56 ± 2.15 cm (range 3 to 14 cm). Based on the bone defect location, proximal 1/3 of the diaphysis in 32 cases (4 in femur, 28 in tibia), middle 1/3 in 129 (31 in femur, 98 in tibia) and distal 1/3 in 121 cases (27 in femur, 94 in tibia). A total of 221 cases underwent single level bone transport procedure and 61 cases were treated by double level bone transport.

The differences of DS, DT, CT, EFT and EFI between single level and double level were statistically significant(*p* < 0.05), whereas they were not statistically significant in the same level(*p* > 0.05). The mean EFI in the double level group is lower than that in the single level group. More details are shown in Table 1, 2 and 3.

**Table 1:** Comparison of the single and double level bone transport group

	Single level group	Double level group	t	*p*-value
DS (cm)	5.84 ± 1.64	9.18 ± 1.78	−13.714	< 0.001
DT(d)	85.69 ± 22.83	70.90 ± 13.87	6.267	< 0.001
CT(d)	254.34 ± 58.55	230.40 ± 39.52	2.986	0.003
EFT(d)	385.15 ± 89.01	340.62 ± 52.18	4.941	< 0.001
EFI(d/cm)	66.54 ± 8.58	38.01 ± 6.52	23.930	< 0.001

Values are presented as mean ± SD. *DS* defect size, *DT* docking time, *CT* consolidation time, *EFT* external fixation time, *EFI* external fixation index

**Table 2:** Comparison of femur and tibia in different group

	Single level group				Double level group			
	Femur	Tibia	t	*p*-value	Femur	Tibia	t	*p*-value
DS (cm)	5.75 ± 1.77	5.87 ± 1.61	−0.437	0.663	9.39 ± 1.47	9.11 ± 1.87	0.520	0.605
DT(d)	85.54 ± 24.27	85.73 ± 22.49	−0.050	0.960	71.64 ± 12.44	70.67 ± 14.40	0.227	0.821
CT(d)	264.02 ± 67.94	251.66 ± 55.59	1.296	0.196	235.29 ± 36.54	228.91 ± 40.66	0.525	0.602
EFT(d)	393.02 ± 92.35	382.97 ± 88.21	0.691	0.490	345.36 ± 46.39	339.17 ± 54.22	0.385	0.701
EFI(d/cm)	67.72 ± 11.26	66.22 ± 7.69	1.073	0.285	37.19 ± 4.65	38.26 ± 7.02	−0.536	0.594

Values are presented as mean ± SD. *DS* defect size, *DT* docking time, *CT* consolidation time, *EFT* external fixation time, *EFI* external fixation index

**Table 3:** Comparison of single and double level group in different bone

	Femur				Tibia			
	SLG	DLG	t	p-value	SLG	DLG	t	p-value
DS (cm)	5.75 ± 1.77	9.39 ± 1.47	−7.021	< 0.001	5.87 ± 1.61	9.11 ± 1.87	−11.738	< 0.001
DT(d)	85.54 ± 24.27	71.64 ± 12.44	2.057	0.044	85.73 ± 22.49	70.67 ± 14.4	4.307	< 0.001
CT(d)	264.02 ± 67.94	235.29 ± 36.54	−3.396	0.001	251.66 ± 55.59	228.91 ± 40.66	2.595	0.010
EFT(d)	393.02 ± 92.35	345.36 ± 46.39	−2.170	0.034	382.97 ± 88.21	339.17 ± 54.2	3.207	0.002
EFI(d/cm)	67.72 ± 11.26	37.19 ± 4.65	9.856	< 0.001	66.22 ± 7.69	38.26 ± 7.02	22.311	< 0.001

Values are presented as mean ± SD. *DS* defect size, *DT* docking time, *CT* consolidation time, *EFT* external fixation time, *EFI* external fixation index, *SLG* single level group, *DLG* double level group

Based on the ASAMI scoring, bony result was excellent in 233 patients (82.62%), good in 32(11.35%), fair in 5(1.77%), poor in 12(4.26%). The functional result was excellent in 136 patients (48.23%), good in 88 (31.21%), fair in 47 (16.67%), poor in 11 (3.90%). The excellent and good rates of bony results was 93.97% (265 of 282), while that of the functional results was 79.43% (224 of 282). (Table 4).

**Table 4:** Results of ASAMI scores

		Excellent	Good	Fair	Poor	Failure
ASAMI	Bone grade	233	32	5	12	–
	Function grade	136	88	47	11	0
ASAMI Criteria						
Bone results						
Excellent: Union, no infection, deformity < 7°, limb length discrepancy (LLD) < 2.5 cm Good: Union plus any two of the following: absence of infection, deformity < 7°, LLD < 2.5 cm. Fair: Union plus any one of the following: absence of infection, deformity < 7°, LLD < 2.5 cm. Poor: Nonunion/refracture/union plus infection plus deformity > 7° plus LLD > 2.5 cm						
Functional results						
Excellent: Active, no limp, minimum stiffness (loss of < 15°knee extension/< 15°ankle dorsiflexion) no reflex sympathetic dystrophy (RSD), insignificant pain. Good: Active, with one or two of the following: limb, stiffness, RSD, significant pain Fair: Active, with three or all of the following: limb, stiffness, RSD, significant pain Poor: Inactive (unemployment or inability to return to daily activities because of injury) Failure: Amputation						

There were 189 problems, 166 obstacles and 406 complications (257 minor complications and 149 major complications) in our study (Table 5). Complications were more prevalent in the single level group (329 complications of 221 patients), whilst it was less in the double level group (77 complications of 61 patients) (Table 5). Most common major complications include joint stiffness (37.58%), delayed union of the docking site (19.46%), axial deviation (18.79%), refracture (8.05%), and muscle contractures (6.04%). Most common minor complications were axial deviation (33.85%), soft tissue incarceration (24.51%), pin-site infection (22.18%), muscle contractures (10.12%), and joint stiffness (4.28%). The average complication rate per patients consists of 0.91 minor and 0.53 major complications (Table 6).

**Table 5:** Difficulties related to the bone transport procedure

	Single level group		Double level group		Total
	Femur	Tibia	Femur	Tibia	
Problem	41	103	14	31	189
Obstacle	47	64	18	37	166
Complication	68	261	23	54	406
Problem: A potential expected difficulty that arises during the distraction or fixation period that is fully resolved by the end of the treatment period by nonoperative means.					
Obstacle: A potential expected difficulty that arises during the distraction or fixation period that is fully resolved by the end of the treatment period by operative means.					
Complication: Any local or systemic intraoperative or perioperative complication, a difficulty during distraction or fixation that remains unresolved at the end of the treatment period, and any early or late posttreatment difficulty.

**Table 6:** Bone transport-related complications

Complications	Minor	Major
Deep pin tract infection or pin loosening	57	3
Muscle contractures	26	9
Joint stiffness	11	56
Axial deviation	87	28
Soft tissue incarceration	63	0
Neurological injury	0	0
Vascular injury	0	0
Delayed consolidation	4	7
Delayed union	9	29
Nonunion	0	5
Refracture	0	12
Total	257	149

The top five complications of our study were pin-site infection (65.96%), axial deviation (40.78%), joint stiffness (23.76%), soft tissue incarceration (22.34%) and delayed union of the docking site (13.48%) (Table 6). Pin tract infection was occurred in most of the patients and managed by daily pin site care and oral antibiotics, 60 patients suffered deep pin tract infection or pin loosening and successfully treated by pin replacement and intravenous antibiotics. Axial deviation occurred in 115 patients, modification of the apparatus or inserting an additional Schanz screw(s) to pull the bone out of its deviated position was required before the end of the treatment. Joint stiffness occurred in 67 cases and treated with physiotherapy along with joint release if needed. Soft tissue incarceration was noted in 63 cases and managed by freshening the bone ends, opening the medullary canal and resection of invaginated soft-tissue. Delayed union was presented in 38 cases and treated by “accordian” technique or bone grafting if it was developed to nonunion.

**Fig. 1:**
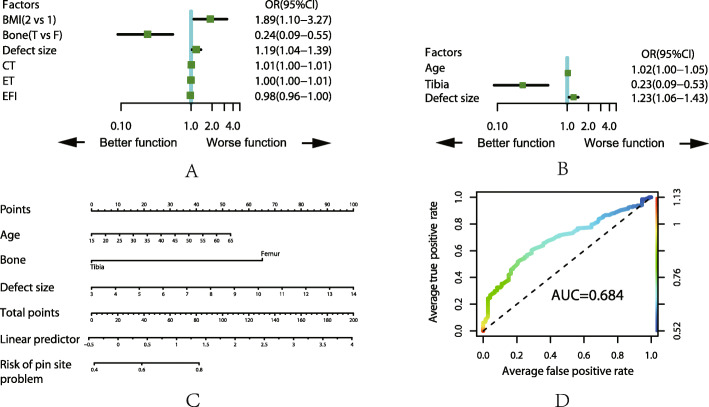
Analysis of the complication of pin site problem. **a** Result of ULRA. **b** Result of MLRA. **c** Nomogram to predict the probability of pin site problem. **d** ROC curves for validating the discrimination power of the nomogram. BMI (1 = underweight, 2 = normal, 3 = overweight, 4 = obesity), Bone (F = femur, T = tibia)

**Fig. 2:**
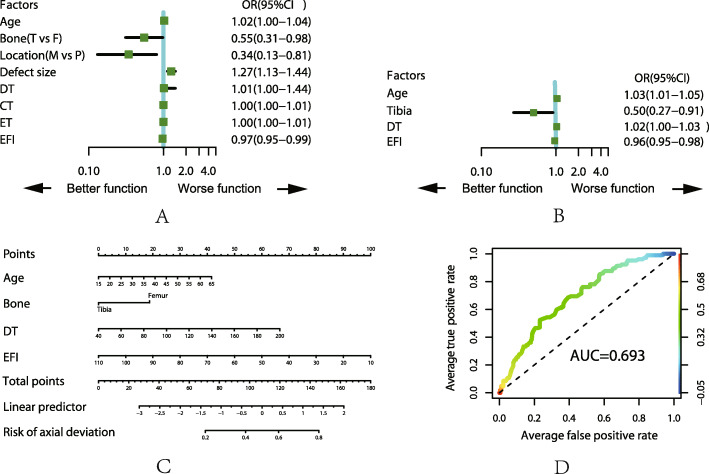
Analysis of the complication of pin site problem. **a** Result of ULRA. **b** Result of MLRA. **c** Nomogram to predict the probability of pin site problem. **d** ROC curves for validating the discrimination power of the nomogram. Bone (F = femur, T = tibia), Location (P = proximal, M = middle)

**Fig. 3:**
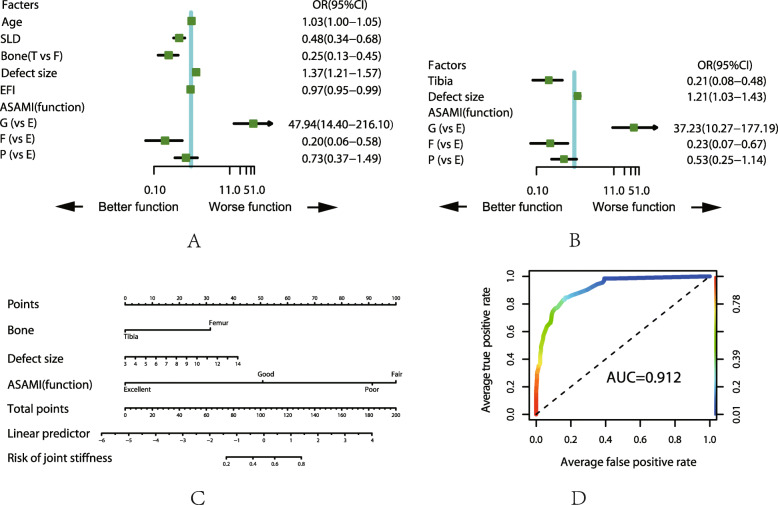
Analysis of the complication of joint stiffness. **a** Result of ULRA. **b** Result of MLRA. **c** Nomogram to predict the probability of pin site problem. **d** ROC curves for validating the discrimination power of the nomogram. Bone (F = femur, T = tibia), ASAMI (E = excellent, G = good, F = fair, P = poor)

**Fig. 4:**
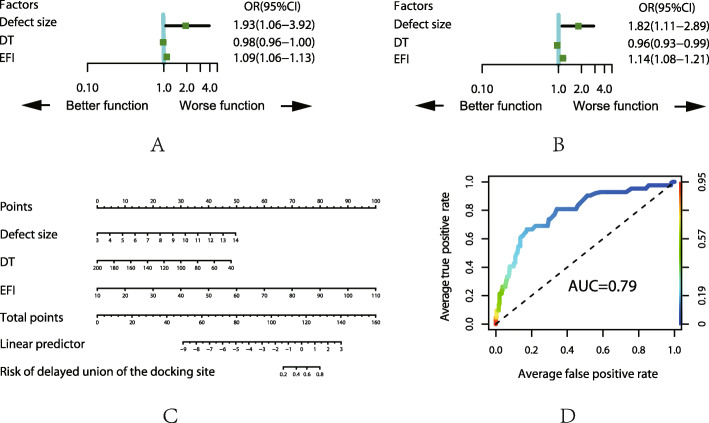
Analysis of the complication of delayed union of the docking site. **a** Result of ULRA. **b** Result of MLRA. **c** Nomogram to predict the probability of pin site problem. **d** ROC curves for validating the discrimination power of the nomogram

For the top five complications, we analyzed those with the related factors by ULRA, MLRA, nomogram and AUC, the details are shown in Fig. 1, 2, 3, 4, and 5. It is generally believed that the model with AUC of 0.50–0.75 is acceptable, and our results are all in this range.

**Fig. 5:**
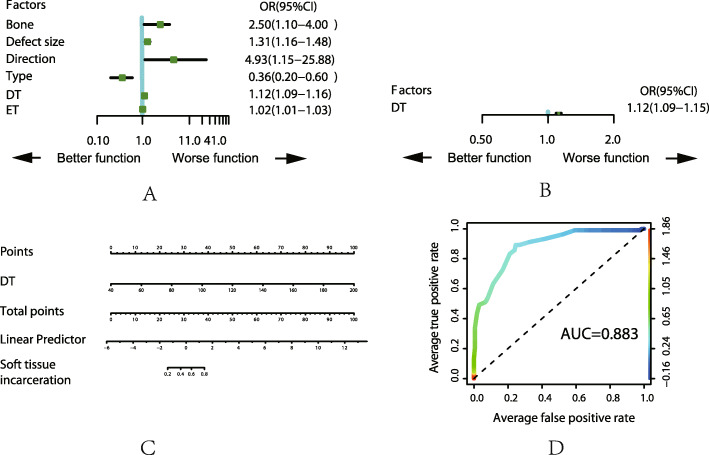
Analysis of the complication of soft tissue incarceration. **a** Result of ULRA. **b** Result of MLRA. **c** Nomogram to predict the probability of pin site problem. **d** ROC curves for validating the discrimination power of the nomogram

## Discussion

Our results showed that double level bone transport can greatly shorten the treatment time compared with single level bone transport. In previous studies [27–31], the mean EFI was 50.0 days/cm (range 43.1 to 58.1 days/cm) in patients treated by single level bone transport, and it is lower than that in our study (66.54 ± 8.58 days/cm). This can be explained by the mechanism of bone defect in our study is mostly caused by posttraumatic osteomyelitis (51.77%) which requires repeated debridement before the initiation of bone transport, the microenvironment for bone regeneration and soft-tissue coverage may destructed, both docking union and regenerate mineralization become time consuming process.

The majority of patients had suffered from various complications during the long treatment period [24, 32, 33]. In the Paley and Maar’s study [34], 19 patients with tibial bone defects were treated with bone transport by Ilizarov method, union was achieved in all cases but 22 minor and 19 major complications were observed, the mean complication rate per patient was 1.15 minor and 1.00 major complications. As for Spieql U et al.^9^, total of 25 patients were included in their study, the patients suffered 22 minor and 13 major complications, and the average complication rate consists of 0.88 minor and 0.52 major complications per patients. Other authors also reported high complication rates in the bone transport procedure [27, 30]. In our study, the average complication rate per patients consists of 0.91 minor and 0.53 major complications.

### Pin track problems

ULRA showed that the presence of pin track problems is less in patient with less BMI, smaller defect size in tibia, shorter CT, ET and EFI. It is due to less soft coverage of tibia in thin patient and shorter time spent in external fixator can significantly reduce the occurrence of pin track problem.

The result of the MLRA showed that less occurrence of pin tract problem in younger patients with smaller tibial defect. Immune system is stronger with better elasticity of local soft tissue in younger patient compared with older one. Besides, more resistance was encounter due to abundant muscle coverage in femur than tibia which can explain the higher occurrence of pin tract problem in femoral bone transport than in tibia.

### Axial deviation

ULRA showed that younger people with bone defect located in the middle 1/3 of tibia suffered less probability of axial deviation. This can be explained by stability of pins are stronger in younger patients (less occurrence of osteoporosis compared with older patient) and muscle tension have less effect in middle 1/3 bone defect during bone transport. The possibility of axial deviation is also less in cases with smaller defect size and shorter DT, CT, ET and EFI which is consisted with the generally accepted theory, the stability of external fixator will be reduced with longer time spend on fixator mountain. The MLRA demonstrated that younger patient, tibial defect, shorter DT and EFI are related with less probability of axial deviation.

### Joint stiffness

ULRA showed that older people with larger femoral defect using double level bone transport tend to have joint stiffness. Additionally, longer EFI lead to prolonged fixation time and patient may reluctant to perform functional exercise which may increase the chance of joint stiffness. As for ASAMI score, the fair and poor grade were not clinically significant. Aforementioned result was also found in the MLRA. Isometric muscle and joint range of motion (ROM) exercise within the tolerance of pain on the second day after surgery is strongly recommended to prevent the occurrence of joint stiffness.

### Delayed union

Both ULRA and MLRA showed that delayed union is more likely to occur in patient with large defect size, longer docking time and EFI. The bone end become ossified due to microvascular occlusion before contact with longer docking time. Moreover, improper application of the external fixator or poor alignment may alter the biomechanical environment required by bone union, in addition, less functional exercise causes less physiological stress stimulation on the bone end, all of these factors contributed to the occurrence of delayed union.

### Soft tissue incarceration

ULRA showed that patient with smaller femoral defect using double level “twin” bone transport is less likely to suffer soft tissue incarceration. Additionally, longer DT and ET also increase the chance of soft tissue incarceration. The result of MLRA demonstrated that only DT is significantly associated with the occurrence of soft tissue incarceration.

In the present study, we used ASAMI scoring system to evaluate the effectiveness of the method of bone transport. The excellent and good rates of bony results was higher than that of functional results and these results were similar to other studies [15, 35, 36].

A total of 12 patients suffered refracture either on docking site or regenerate region. Among them, 4 cases were due to early removal of external fixator and 8 cases were due to a fall injury (5 cases) or a car accident (3 cases) after the removal of the protective plaster and all of them achieved union by open reduction and plate fixation.

Ilizarov method is inadvisable for the socially disadvantaged patients, especially those with poorly managed mental illness or those without a supportive family environment, mainly due to prolonged treatment period associated with potential complications. A number of factors may contribute to the occurrence of complications during the bone transport procedure. Based on our retrospective study, age, BMI, defect size, injured bone, location of bone defect, type of bone transport, direction of bone transport, docking time, regenerate consolidation time, external fixation time and external fixation index are statistically significantly associated with the occurrence of complications; age, defect size and external fixation time are the prominent predictors of complications.

The present study had several limitations. First, considering its retrospective nature and relatively small sample size, prudent attitude should be adopted regarding the interpretations of our bone and functional outcomes. Second, longer follow-up time is necessary to better evaluate the clinical efficacy. Third, further investigations, especially multi-centered trails with a larger sample size should be conducted to overcome the limitations of our study.

## Conclusion

Our study presents the results of 282 consecutive cases using single or double level bone transport with particular reference to the complications and its related factors. Bone transport is a reliable method for reconstruction of bone defects in the femur and tibia caused by variety of reasons. Awareness of predictable complications is beneficial to prevent or early detection of the expected complication which can improve the risk-benefit balance. Regular follow-up, prudent patient selection, shorter fixator’s time and particular attention during the process are necessary to reduce the related complications.

## Data Availability

The datasets analysed during the current study are available from the corresponding author on reasonable request.

## Abbreviations

ASAMIA: Association for the Study and Application of the Method of Ilizarov criteria
DS: Defect size
DRL: Distraction regenerate length
DT: Docking time
CT: Regenerate consolidation time
EFT/ET: External fixation time
EFI: External fixation index
ROM: Range of motion
ESR: Erythrocyte sedimentation rate
CRP: C-reactive protein
AUC: Area under the curve
ROC: Receiver operating characteristic
ULRA: Univariate logistic regression analysis
MLRA: Multivariate logistic regression analysis
